# Adherence to the American Academy of Pediatrics Health Supervision Guidelines for Children With Down Syndrome in a Tertiary Care Institution in Northeastern Colombia

**DOI:** 10.7759/cureus.93426

**Published:** 2025-09-28

**Authors:** Silvia N Suárez-Mantilla, Martha L Africano-Leon, Victor M Mora-Bautista, Diana C Vergara-Arenas, Angie T Bustos-López, Sergio Serrano-Gomez

**Affiliations:** 1 Pediatrics, Universidad Industrial de Santander, Bucaramanga, COL; 2 Pediatrics, Clinica Materno Infantil San Luis, Bucaramanga, COL

**Keywords:** adherence, adherence to guidelines, colombia, down's syndrome, health care disparities, health maintenance, health supervision, health surveillance, primary care, trisomy of 21

## Abstract

Health surveillance of children with Down syndrome is often insufficient. We assessed adherence to the 2011 American Academy of Pediatrics (AAP) health supervision guidelines at a tertiary pediatric care institution in northeastern Colombia. This descriptive, cross-sectional study analyzed electronic medical records of 124 children under 13 years (59.7% were males, and 66% resided in a metropolitan area) seen between 2012 and 2023. Overall adherence was 57%. Adherence rates varied by age: high in neonates and school-aged children (>80%), moderate in infants and preschoolers (68%), and low in toddlers (39%). While most children underwent echocardiogram (99%), complete blood count (90%) and karyotype testing (78%) within the first month, fewer received timely hearing evaluations by six months (60%) or ophthalmologic evaluation by six months (34%). Significant non-adherence occurred during the first year and persisted through early childhood, highlighting the need to identify barriers and improve guideline compliance.

## Introduction

Down syndrome (DS), or trisomy 21, is the most common chromosomal disorder, with an estimated incidence ranging from one in 319 to one in 1,000 live births ​[1]. Children with DS have an increased predisposition to various medical conditions, including congenital heart defects, neurodevelopmental disorders, and musculoskeletal, respiratory, thyroid, and hematologic abnormalities, among others​ [2]. 

In recent decades, life expectancy in this population has significantly increased from 25 years in 1983 to approximately 60 years today ​[3]​. This progress is largely attributed to improved access to comprehensive and timely medical care. Within this context, preventive practices have played a key role in reducing severe complications, as individuals with DS require lifelong medical follow-up ​[4]. 

The use of anticipatory guidelines is essential not only for the early detection of abnormalities but also for promoting the development of specialized healthcare services, strengthening support networks, and ultimately improving patients' quality of life and life expectancy. 

To support these efforts, organizations such as the American Academy of Pediatrics (AAP) have developed anticipatory management guidelines, including the Health Supervision for Children With Down Syndrome, first published in 1994 ​[5]. This document has been updated in 2001, 2011, and most recently in 2022, with the aim of incorporating new scientific knowledge to better address medical needs and promote a more individualized approach ​[2,6-8]. However, despite the time that has passed since its initial publication, evidence suggests that these clinical recommendations are still not adequately implemented in many healthcare settings ​[9]. 

Therefore, this study aimed to assess adherence to the 2011 Health Supervision for Children with Down Syndrome guidelines to identify priority areas for improving care for individuals with this condition [8].

## Materials and methods

Study design and settings

A cross-sectional study using historical data collected between January 2012 and December 2023, was conducted from January to December 2024 to assess adherence to the 2011 Health Supervision for Children with Down Syndrome guidelines by the AAP in a tertiary care institution in northeastern Colombia. The study was approved by the Clínica Materno Infantil San Luis Research Ethics Committee (approval 23072024).

Eligible subjects had a confirmed diagnosis of Down syndrome, were younger than 13 years of age, and had attended at least one medical follow-up visit during the study period. Subjects were excluded if their medical records were missing or incomplete.

Adherence to the 2011 Health Supervision for Children with Down Syndrome guideline [8] was assessed both globally and stratified by age group (les than one month, one to 12 months, one to five years, and five to 12 years), according to the age distribution outlined in the guideline, as well as by the specific diagnostic tests performed. Patients older than 12 years were not included, since the guideline classifies this group as 13 to 21 years, and this pediatric clinic only provides care for individuals under 18 years of age.

Data collection and analysis

Electronic medical records were reviewed to identify eligible subjects. The search was conducted using the following International Classification of Diseases, 10th Revision (ICD-10) codes: Q900, Q901, Q902, and Q909 [10]. A total of 124 eligible subjects were identified and included in the analysis. All data were extracted from the hospital's internal electronic medical record system. Each record was assigned a unique code and contained no personally identifiable information. Data were stored in a password-protected database. The variables considered are detailed in Table 1 .

**Table 1:** Criteria for adherence to health supervision guideline for children with Down syndrome. TSH: Thyroid-stimulating hormone. Adapted from the 2011 version of the Health Supervision Management Guideline for Children with Down Syndrome ​[8]​

Criteria of adherence	<1 month	1–12 months	1–5 years	5–12 years
Karyotype	X			
TSH at birth	X			
TSH at 6 months		X		
TSH at 12 months		X		
Weight and height screening		X	X	X
Echocardiogram	X			
Ophthalmology before 6 months		X		
Ophthalmology between 6 months and 13 years		X	X	X
Complete blood count in the first month	X			
Annual complete blood count		X	X	X
Hearing screening before 6 months	X	X		
Hearing screening between 6 months and 4 years		X	X	
Total	5	8	4	3

Adherence levels were defined according to the thresholds proposed by Ortega and Vargas (2014), based on the instrument developed by Bonilla and Gutiérrez (2007): adherence was considered present when equal to or greater than 80%; a risk of non-adherence was defined as between 60% and 79%; and non-adherence as below 60% [11-13].

Statistical analysis

All analyses were performed using STATA version 16 (StataCorp., College Station, TX, USA). Quantitative variables were described using measures of central tendency and dispersion (mean and standard deviation), as all continuous variables followed a normal distribution as assessed by the Shapiro-Francia test. Qualitative variables were described using absolute and relative frequencies. 

## Results

Sociodemographic characteristics

A total of 124 subjects were evaluated, of whom 74 (59.7%) were males. The mean age was 19.7 months (SD ±28.87; range 1-132 months). Regarding health insurance, 68 (54.8%) were enrolled by the contributory regime, and 82 (66.1%) resided in the metropolitan area of Bucaramanga. All sociodemographic data are presented in Table 2.

**Table 2:** Sociodemographic characteristics of subjects with Down syndrome

Sociodemographic Characteristics	n	%
Sex		
Female	50	40.3
Male	74	59.7
Age groups		
Less than or equal to 1 month	46	37.1
1–12 months	30	24.2
1–5 years	35	28.2
5–12 years	13	10.5
Place of residence		
Urban	82	66.1
Rural	42	33.9
Type of health insurance		
Contributory	68	54.8
Subsidized	47	37.9
Special regime	9	7.3
Final condition		
Alive	116	93.5
Deceased	8	6.5

Overall adherence to clinical recommendations

**Figure 1:**
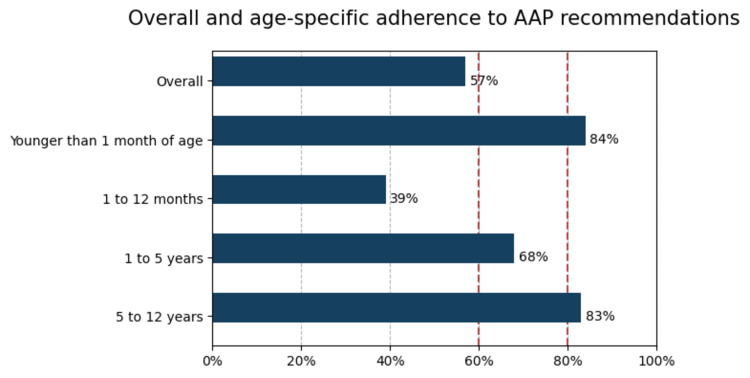
Overall and age group-specific adherence to AAP recommendations AAP: American Academy of Pediatrics Red dashed lines represent adherence levels of 60% and 80%, respectively.

Global adherence, according to the criteria outlined in Table 1, averaged 57% (SD ±16.43) and was classified as being at non-adherence. Figure 1 presents this result in comparison to adherence rates overall and by the age groups defined in the guideline. 

Adherence to clinical recommendations by age group and specific interventions 

Adherence by age group, based on the 2011 Health Supervision Management Guideline for Children with Down Syndrome [8] as summarized in Table 1, varied across the study population. Among infants younger than one month, the mean number of fulfilled criteria was 4.2/5 (SD ±0.78), equivalent to 84% (adherent). In the one to 12 month group, the mean was 3.16/8 (SD ±1.87), corresponding to 39% (non-adherent). In children aged one to five years, adherence averaged 2.71/4 criteria (SD ±1.06), equivalent to 68% (at risk of non-adherence). In those aged five to 12 years, the mean was 2.5/3 (SD ±0.65), corresponding to 83% (adherent).

Clinical screening and follow-up results

**Figure 2:**
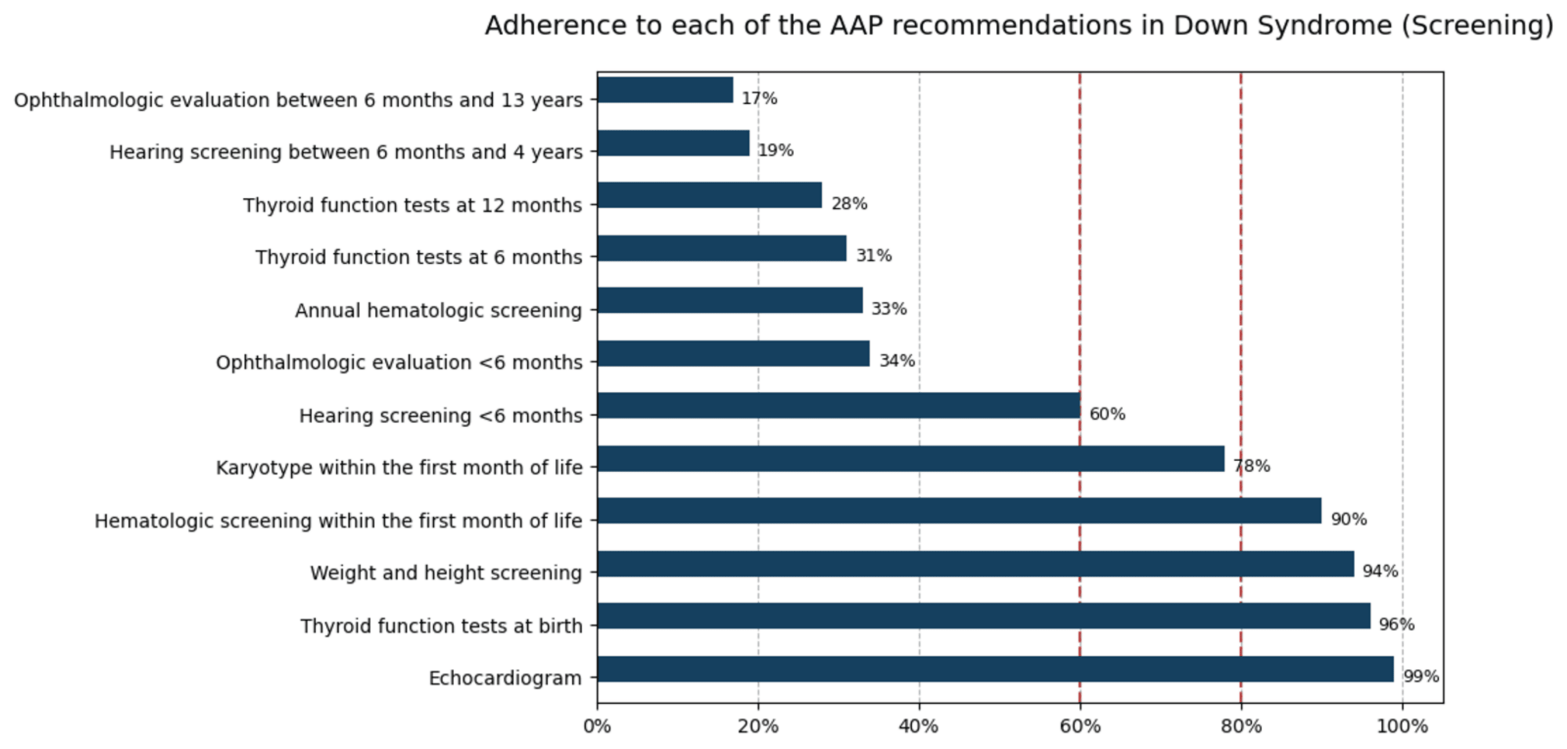
Adherence to each of the recommendations in the AAP guidelines AAP: American Academy of Pediatrics Red dashed lines represent adherence levels of 60% and 80%, respectively.

Figure 2 shows the clinical screening and follow-up results. Compliance with only 50% of the assessed recommendations was observed. Karyotype testing was performed during the first month of life in 97 cases (78%). Results were available for 31 patients, with free trisomy being the most frequently observed chromosomal abnormality (27 cases, 87.1%). Thyroid function tests were performed at birth in 119 children (96%). Follow-up testing declined substantially, with only 38 (31%) undergoing evaluation at six months and 35 (28%) at 12 months of age. Hypothyroidism was identified in 27 cases (22%).

Hearing screening was completed in 74 cases (60%) before six months of age, but only 24 (19%) underwent testing between six months and four years. Documented hearing abnormalities were found in six records (4.9%), two of whom had profound deafness. Echocardiography adherence was notably high, with 123 participants (99%) undergoing the procedure during the first month of life. Congenital heart disease was diagnosed in 96 patients (77.4%), most commonly atrial septal defect (60 cases, 48.4%), followed by ventricular septal defect (47 cases, 37.9%) and atrioventricular canal defect (30 cases, 24.2%). Surgical intervention was required in 32 cases (25.8%).

Ophthalmologic evaluation was performed in 42 subjects (34%) before six months of age and in 22 (17%) between six months and 13 years. Ocular abnormalities were documented in 14 evaluated records (11.3%), the most frequent being astigmatism. Hematologic screening was conducted in 112 patients (90%) during the first month of life, although annual follow-up was recorded in only 41 (33%). Acute lymphoblastic leukemia was diagnosed in four patients (3.2%). Nutritional screening, including weight and height, was documented in 94% of medical records. Of these, 57 (46%) demonstrated adequate nutritional status, 29 (23.4%) were at risk of malnutrition, 13 (10.5%) had moderate acute malnutrition, and 13 (10.5%) had severe acute malnutrition.

## Discussion

This study represents the first reported analysis in Latin America examining adherence to the 2011 AAP health supervision guidelines for DS [8], as identified in the literature review.

In this study, adherence to AAP recommendations was highest during the first month of life. However, adherence declined markedly between one and 12 months of age. Similarly, O'Neill et al. found that children under 13 years of age exhibited significantly higher adherence rates to AAP recommendations (86-89%). In contrast, adolescents aged 13 and older had an adherence rate of only 60%. While these findings cannot be directly compared due to different study populations, they support the hypothesis that younger patients show greater adherence to established recommendations [14].

This pattern may reflect that neonatal care follows structured hospital protocols delivered by specialized teams. In contrast, after the neonatal period - particularly between one and 12 months - outpatient follow-up relies heavily on the individual clinical judgment of healthcare providers and may lack systematic tools such as clinical reminders or templates within electronic health records. Previous studies have identified this as a key barrier, suggesting that the absence of automated systems integrating recommendations into routine clinical workflow may significantly contribute to lower adherence [15].

Regarding adherence by type of screening, echocardiography showed the highest compliance rate (99%), consistent with its recommendation within the first month of life. This high level of adherence is particularly relevant, given that children with DS and congenital heart disease face up to a 25-fold higher mortality risk compared to children without DS. However, screening strategies have led to significant improvements in survival rates over recent decades [13,14]. This finding is consistent with reports from other studies: Livingstone et al. reported 98% adherence, Rojnueangnit et al. 96%, and Schoonraad et al. 79% [9,16,17].

With regard to thyroid disease screening, neonatal adherence was high; however, fewer than half of the patients received complete thyroid follow-up, despite hypothyroidism being the second most frequent pathology after congenital heart disease in the DS patients evaluated. This pattern is consistent with studies conducted in the United States and Ireland, which report overall adherence rates ranging from 34% to 61%, reflecting a significant gap in postnatal follow-up [18-21].

Ophthalmologic screening showed the lowest compliance rate in this study, limiting the available data on the prevalence of ocular abnormalities, which should therefore be interpreted with caution [21]. In general, low adherence to AAP recommendations was observed for hearing, ophthalmologic, and hematologic screening protocols.

Based on these findings, the absence of structured local follow-up programs may explain this non-adherence for DS patients, as well as the lack of clinical guideline compliance checklists. During the neonatal period, better follow-up may occur due to care received in neonatal units. During the first year of life, the increasing number of monitoring requirements may contribute to decreased adherence. Subsequently, as monitoring demands decrease, adherence rates may improve.

While the incorporation of management guidelines for Down syndrome has demonstrably improved morbidity and mortality outcomes, our study reveals persistently low adherence across multiple domains. The long-term implications of inadequate guideline implementation are substantial, as demonstrated by Baksh et al., who reported significantly elevated risks among individuals with Down syndrome for dementia (incidence rate ratio (IRR) 16.60, 95% CI 14.23-19.37), hypothyroidism (IRR 7.22, 95% CI 6.62-7.88), obstructive sleep apnea (IRR 4.45, 95% CI 3.72-5.31), and hematological malignancy (IRR 3.44, 95% CI 2.58-4.59). These findings underscore a critical association between suboptimal guideline adherence and increased morbidity burden, with consequent implications for healthcare costs and patient outcomes [22].

The influence of residence and socioeconomic conditions on healthcare access deserves particular attention. A considerable proportion of our study population traveled from rural areas or outside the city for medical consultations, reflecting significant health service inequities. This finding aligns with a Chilean study demonstrating delays in skill acquisition among children with Down syndrome, where those from higher socioeconomic strata showed delays only in language development, while children from lower socioeconomic backgrounds exhibited significant deficits across cognitive, language, and socio-emotional domains [23]. Similarly, Varshney et al. identified financial barriers as contributors to incomplete healthcare trajectories for patients with Down syndrome, linking these obstacles to insurance limitations and constrained provider resources and time [24].

These findings underscore the need for context-specific public health policies, including tailored guidelines for managing patients with Down syndrome that address the socioeconomic realities of our population. Additionally, strengthening healthcare professional training and education is essential to ensure proper guideline implementation. Strict adherence to established guidelines is therefore essential to mitigate disease burden and optimize care quality in this vulnerable population. Future research should evaluate the actual impact of these inequities on guideline adherence and health outcomes.

Limitations 

This study has several limitations related to its retrospective, observational design, including exclusive reliance on medical records, which may contain incomplete, inconsistent, or erroneous information, potentially affecting data accuracy. Differences in clinical judgment among providers over the study period, as well as changes in insurance coverage or place of residence, may also have influenced the consistency of follow-up.

## Conclusions

This study demonstrated high adherence to DS supervision guidelines during the first month of life, followed by significant non-adherence to key screening components throughout the first year and continued suboptimal adherence during early childhood. Adherence improved in later years. These findings highlight the urgent need for structured institutional follow-up programs and implementation of existing clinical checklists. Additionally, development of national guidelines adapted to local health systems and available resources is essential.
